# Calibrated comparison of SARS-CoV-2 neutralizing antibody levels in response to protein-, mRNA-, and vector-based COVID-19 vaccines

**DOI:** 10.1038/s41541-022-00455-3

**Published:** 2022-02-18

**Authors:** Michael Karbiener, Maria R. Farcet, Andreas Zollner, Taisei Masuda, Mitsuhiro Mori, Alexander R. Moschen, Thomas R. Kreil

**Affiliations:** 1 Global Pathogen Safety, Takeda Manufacturing Austria AG, Vienna, Austria; 2 Christian Doppler Laboratory for Mucosal Immunology, Johannes Kepler University Linz, Linz, Austria; 3 Department of Medicine, Division of Internal Medicine 1 (Gastroenterology and Hepatology, Endocrinology and Metabolism), Medical University of Innsbruck, Innsbruck, Austria; 4 Japan Development, Global Vaccine Business Unit, Takeda Pharmaceutical Company Limited, Osaka, Japan; 5 Department of Internal Medicine 2 (Gastroenterology and Hepatology, Endocrinology and Metabolism, Nephrology, Rheumatology), Johannes Kepler University Linz, Linz, Austria

**Keywords:** Vaccines, Drug development

## Abstract

SARS-CoV-2 neutralizing antibodies have been suggested to reflect the efficacy of COVID-19 vaccines. This study reports the direct comparison of the SARS-CoV-2 neutralizing antibody response elicited by a protein- (NVX-CoV2373), an mRNA- (Comirnaty), and a vector-based (Vaxzevria) COVID-19 vaccine, calibrated against the WHO international SARS-CoV-2 antibody standard, and further supports the use of neutralizing antibody levels as a correlate of protection.

Since late 2019, *Severe acute respiratory syndrome coronavirus 2* (SARS-CoV-2) has rapidly spread around the globe, and the resulting coronavirus disease 2019 (COVID-19) pandemic has had unprecedented impact on healthcare systems, economics, and social interactions. At similarly unprecedented speed, the development, clinical investigation, and regulatory assessment of COVID-19 vaccines have been pursued^1^, resulting in emergency use authorization (EUA) of several vaccines just about one year after the virus had initially been described. The first two formulations licensed in Western countries—Comirnaty (BioNTech-Pfizer) and Spikevax (Moderna)—employed an mRNA vaccine technology, while shortly thereafter, two vaccines based on recombinant adenoviral vectors—Vaxzevria (AstraZeneca) and Janssen COVID-19 Vaccine (Janssen)—received regulatory approvals in the European Union (EU) or the United States (US); all of these vaccines were characterized by high efficacy across gender, age groups, and ethnicities^2–5^.

Another large group of COVID-19 vaccine candidates employs the biotechnological production of immunogenic viral proteins or protein subunits, in case of SARS-CoV-2 the spike (S) protein or its receptor-binding domain^1^. One of these candidates is NVX-CoV2373 (TAK-019; Novavax), a recombinant nanoparticle vaccine for which safety and immunogenicity^6^ and subsequently high efficacy^7^ have been demonstrated. Compared to mRNA vaccines, long-term storage above freezing is a beneficial characteristic that is especially important for the supply of low- and middle-income countries. Correspondingly, as the first protein-based vaccine, NVX-CoV2373 has recently been granted EUA in Indonesia^8^ and the Philippines^9^, as well as a conditional marketing authorization in the EU^10^.

Large vaccination campaigns have meanwhile substantiated the effectiveness of the aforementioned mRNA- and vector-based vaccines, especially with respect to severe COVID-19 and thus COVID-19-related death. With respect to further vaccine candidates currently in the pipeline, there is a demand for estimating such performance indicators upfront, as the ultimately expected global reduction in case numbers (due to the already licensed vaccines) and ethical considerations (avoiding placebo groups when vaccines are already standard care) argue against large phase 3 efficacy studies^11^. Not unexpectedly, the levels of SARS-CoV-2 neutralizing antibodies (nAbs), and antibodies binding to the S protein, have recently been identified as promising correlates of protection^12,13^, i.e., measuring SARS-CoV-2 antibodies in serum samples of vaccinees enables to predict the risk of developing COVID-19. However, while the clinical evaluation of vaccine candidates regularly includes such antibody readouts, the heterogenous design of the underlying virus neutralization and binding assays limits the quantitative comparison of the primary result (e.g., the neutralization titer, or arbitrary binding units) across distinct studies. These technical hurdles can be alleviated by the incorporation of an international standard, which has recently been made available to study SARS-CoV-2 antibody-containing samples^14^. Indeed, this international standard has recently been employed for analyses of vaccinee sera from the two mRNA- and two vector-based vaccines mentioned above to predict an overall protective level of S protein-binding antibodies^15^. Further, using a pseudovirus neutralization assay, Gilbert et al. found that anti-SARS-CoV-2 potencies of 8 and 140 international units per milliliter (IU/mL) correspond to 70 and 90% efficacy of the Spikevax vaccine, respectively^16^. By additional comparison to a similar study on Vaxzevria recipients’ sera^17^, the authors also showed an encouragingly congruent quantitative relationship (4 and 83 IU/mL corresponding to 70 and 90% efficacy, respectively), despite the different vaccine platforms and pseudoviral assays.

In the present study, we made use of the first international SARS-CoV-2 antibody standard and directly compared the nAb levels in response to a protein-, an mRNA- and a vector-based vaccine. All study subjects were confirmed to have been initially seronegative (no detectable SARS-CoV-2 neutralization for samples obtained at the day of first vaccination). Samples from individuals vaccinated either with NVX-CoV2373 (*n* = 30), Comirnaty (*n* = 35) or Vaxzevria (*n* = 12) were collected 15–32 days after complete immunization (2 doses of the respective vaccine; Table 1). Average post-vaccination anti-SARS-CoV-2 potency was 548 IU/ml, 557 IU/ml, and 202 IU/ml for recipients of NVX-CoV2373, Comirnaty, and Vaxzevria, respectively (Fig. 1). ANOVA (*P* = 0.004) and post-hoc pairwise comparisons confirmed that mean SARS-CoV-2 nAb levels were equivalent for NVX-CoV2373 and Comirnaty groups (adjusted *P* value: 0.998) and significantly lower for the Vaxzevria group (adjusted P values: NVX-CoV2373 vs. Vaxzevria: 0.007; Comirnaty vs. Vaxzevria: 0.005). SARS-CoV-2 nAb levels were insignificantly affected by donor age (*P* = 0.122) and gender (*P* = 0.768).

**Table 1:** Demographic characteristics of study groups and summary data of serum SARS-CoV-2 neutralizing antibody levels.

vaccine (manufacturer)	NVX-CoV2373 (Novavax)	Comirnaty (Pfizer-BioNTech)	Vaxzevria (AstraZeneca)
n	30	35	12
mean donor age (min−max) [yr]	53 (20–76)	39 (23–62)	44 (27–59)
female	43%	66%	75%
male	57%	34%	25%
ethnicity	Asian	White	White
mean time since 1st vaccination ± SD [d]	36 ± 0	49 ± 4	106 ± 3
mean time since 2nd vaccination ± SD [d]	15 ± 0	27 ± 3	26 ± 4
geometric mean (lower; upper 95% CI) of anti-SARS-CoV-2 potency [IU/ml]	548 (368; 818)	557 (428; 725)	202 (107; 382)

**Fig. 1:**
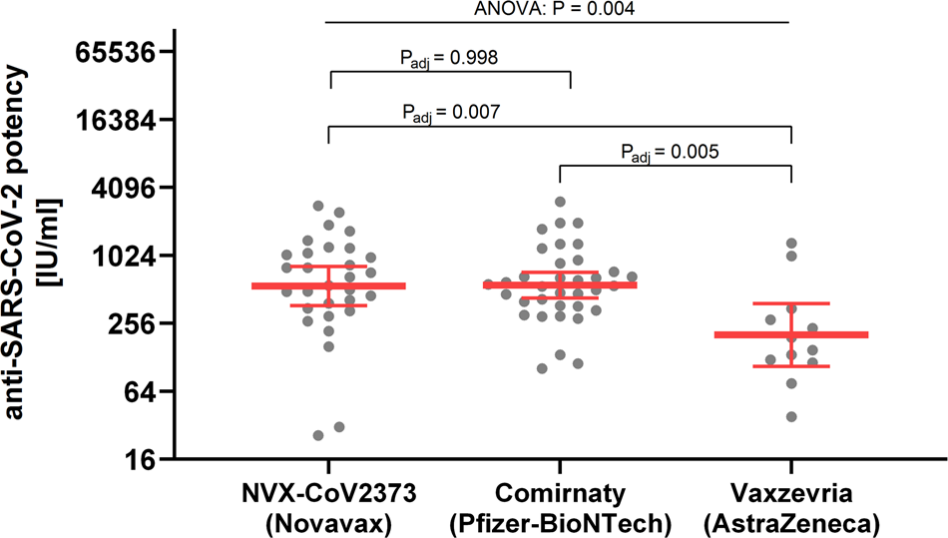
SARS-CoV-2 neutralizing antibody levels in initially seronegative, twice vaccinated individuals. Serum samples were obtained 15 to 32 days after complete SARS-CoV-2 immunization, i.e., 2 doses of either NVX-CoV2373 (*n* = 30), Comirnaty (*n* = 35), or Vaxzevria (*n* = 12), and analyzed by live virus neutralization assay. Anti-SARS-CoV-2 potency is given as IU/ml, i.e., relative to the first WHO international SARS-CoV-2 antibody standard (NIBSC code 20/136). Individual samples are shown as grey dots and overlaid by geometric mean ± 95% confidence interval. Statistical analysis (one-way ANOVA with Tukey’s multiple comparisons test) was conducted on ln-transformed data.

Previously, the explanatory power of SARS-CoV-2 nAbs with respect to vaccine efficacy has been investigated by an indirect approach, i.e., via normalization of neutralization titers to cohorts of convalescent (post-COVID-19) individuals analyzed in parallel^12,13^. However, such cohorts are subject to considerable variation (e.g., due to different parameters that define convalescence, due to diverging fractions of individuals that had suffered from severe versus mild disease, or sample size), as is the sequence of mathematical operations for normalization. Thus, while Khoury et al. found the average SARS-CoV-2 nAb levels induced by Vaxzevria to be lower than average post-COVID-19 levels^12^, Earle et al. found slightly higher mean anti-SARS-CoV-2 potency of the vaccine^13^. The latter result is similar to our own data, as the mean vaccine-induced SARS-CoV-2 nAb level of 202 IU/ml (Table 1) is above the mean (140 IU/ml) of a post-COVID-19 group analyzed in one of our earlier studies^18^. The accuracy of the earlier used comparison to convalescent plasma for normalization must therefore be considered limited.

In contrast, the results of the present study are derived from the same assay and provide a first direct comparison of the SARS-CoV-2 nAb response between three COVID-19 vaccines based on different immunogenic principles. Comparable anti-SARS-CoV-2 potency in sera of NVX-CoV2373 and Comirnaty recipients and the slightly lower levels in response to Vaxzevria are in line with previously published levels of vaccine efficacy^2,4,7^, directionally confirm the previous, more indirect approaches^12,13^ and lend further support to the notion that neutralizing antibody responses represent a suitable correlate of protection. Between the three vaccination groups, there is a somewhat diverging mean time between second vaccination and sample collection (Table 1), which is a limitation of our study. However, it should be noted that the peak immune response (and hence the achievement of a ‘fully vaccinated’ status) is generally considered to commence two weeks after the second shot (i.e., when serum samples from the NVX-CoV2373 group were obtained) and antibody waning occurs over several weeks to months rather than days (the latter being the difference in sampling between our three groups). Further, while distinct ethnicities might be a confounding factor of the present study, it should be noted that diverging COVID-19 vaccine efficacy between Asian and White vaccine recipients has not been reported^2,4^. Most importantly, the calibration of results against the first international SARS-CoV-2 antibody standard^14^ allows for an objective comparison beyond the present study population.

## Methods

### SARS-CoV-2 neutralization assay

Detection of SARS-CoV-2 neutralizing antibodies employed live SARS-CoV-2 (strain BetaCoV/Germany/BavPat1/2020) and was based on microscopic readout of cytopathic effects on Vero cells^19^. Briefly, human serum samples were prediluted 1:5–1:40 with cell culture medium followed by serial dilution in twofold steps. Sample dilutions were combined with an equal volume of virus stock at 10^3.0^ tissue culture infectious doses 50% per milliliter (TCID_50_/mL) and incubated for 150 ± 15 min before titration on Vero cells (Cat. no. 84113001, ECACC, Porton Down, Salisbury, UK) in eight-fold replicates per dilution. The virus-induced cytopathic effect was determined after 5–7 days of incubation (36 °C; humidified atmosphere with 5% CO_2_). The 50% neutralization titer, i.e., the reciprocal sample dilution resulting in 50% virus neutralization (NT_50_) was determined using the Spearman-Kaerber formula. The neutralization assay included several validity criteria, i.e., confirmatory titration of input virus infectivity, cell viability, and neutralization testing of an internal reference standard, all of which had to comply with defined ranges. Further, the internal standard was calibrated against the First WHO International Standard for anti-SARS-CoV-2 immunoglobulin (human; NIBSC code: 20/136)^14^ to enable the quantification of anti-SARS-CoV-2 potency in international units per milliliter (IU/ml). Data from 28 subjects of the Comirnaty group has been included in a previous study^20^.

### Ethics

The study was performed in accordance with applicable regulations, policies, and procedures, and the authors’ institutions required informed consent that was obtained from all study subjects. For Comirnaty and Vaxzevria groups, all subjects received two vaccinations according to EMA approval. For NVX-CoV2373, samples from 30 participants who received two vaccinations of NVX-CoV2373/TAK-019 (5 µg of a recombinant nanoparticle spike protein plus 50 µg of Matrix-M adjuvant; with 21 days apart) of an ongoing phase 1/2 clinical trial in Japan (TAK-019-1501 study, ClinicalTrials.gov Identifier: NCT04712110) were randomly selected.

### Data preparation and statistical analyses

Basic statistical calculations for normalization of SARS-CoV-2 neutralization titers and conversion into IU/ml were conducted using MS Excel (v2102; Microsoft, Redmont, WA, US). For hypothesis testing, SARS-CoV-2 nAb levels (IU/ml) were ln-transformed. Graphical illustration and statistical analyses for the influence of distinct vaccines were done using GraphPad Prism (v8.1.1; GraphPad Software, San Diego, CA, US; one-way ANOVA and Tukey’s multiple comparisons test). The influence of donor age and gender was assessed using Minitab (v17.3.1; Minitab, LLC, State College, PA, US; general linear model). All P values are 2-sided and an alpha level of 0.05 is used.

### Reporting summary

Further information on research design is available in the Nature Research Reporting Summary linked to this article.

## Supplementary information

Reporting Summary

## Data Availability

The data that support the findings of this study are available from the corresponding author upon reasonable request.
