# Supplementary material for: Calibrated comparison of SARS-CoV-2 neutralizing antibody levels in response to protein-, mRNA-, and vector-based COVID-19 vaccines
Source: NPJ Vaccines. 2022 Feb 18;7:22. doi: 10.1038/s41541-022-00455-3 (PMC8857217; doi:10.1038/s41541-022-00455-3)
Supplement: Supplementary file 1 — Reporting Summary [file 41541_2022_455_MOESM1_ESM.pdf]

nature portfolio

Corresponding author(s): Thomas R. Kreil

Last updated by author(s): Jan 13, 2022

Reporting Summary

Nature Portfolio wishes to improve the reproducibility of the work that we publish. This form provides structure for consistency and transparency in reporting. For further information on Nature Portfolio policies, see our Editorial Policies and the Editorial Policy Checklist.

Statistics

For all statistical analyses, confirm that the following items are present in the figure legend, table legend, main text, or Methods section.

n/a	Confirmed	
☐	☒	The exact sample size (*n*) for each experimental group/condition, given as a discrete number and unit of measurement
☒	☐	A statement on whether measurements were taken from distinct samples or whether the same sample was measured repeatedly
☐	☒	The statistical test(s) used AND whether they are one- or two-sided<br>*Only common tests should be described solely by name; describe more complex techniques in the Methods section.*
☐	☒	A description of all covariates tested
☐	☒	A description of any assumptions or corrections, such as tests of normality and adjustment for multiple comparisons
☐	☒	A full description of the statistical parameters including central tendency (e.g. means) or other basic estimates (e.g. regression coefficient) AND variation (e.g. standard deviation) or associated estimates of uncertainty (e.g. confidence intervals)
☐	☒	For null hypothesis testing, the test statistic (e.g. *F*, *t*, *r*) with confidence intervals, effect sizes, degrees of freedom and *P* value noted<br>*Give P values as exact values whenever suitable.*
☒	☐	For Bayesian analysis, information on the choice of priors and Markov chain Monte Carlo settings
☒	☐	For hierarchical and complex designs, identification of the appropriate level for tests and full reporting of outcomes
☒	☐	Estimates of effect sizes (e.g. Cohen's *d*, Pearson's *r*), indicating how they were calculated

Our web collection on statistics for biologists contains articles on many of the points above.

Software and code

Policy information about availability of computer code

Data collection: n.a.

Data analysis: n.a.

For manuscripts utilizing custom algorithms or software that are central to the research but not yet described in published literature, software must be made available to editors and reviewers. We strongly encourage code deposition in a community repository (e.g. GitHub). See the Nature Portfolio guidelines for submitting code & software for further information.

Data

Policy information about availability of data

All manuscripts must include a data availability statement. This statement should provide the following information, where applicable:

- Accession codes, unique identifiers, or web links for publicly available datasets
- A description of any restrictions on data availability
- For clinical datasets or third party data, please ensure that the statement adheres to our policy

The data that support the findings of this study are available from the corresponding author upon reasonable request.

Field-specific reporting

Please select the one below that is the best fit for your research. If you are not sure, read the appropriate sections before making your selection.

☒ Life sciences ☐ Behavioural & social sciences ☐ Ecological, evolutionary & environmental sciences

For a reference copy of the document with all sections, see nature.com/documents/nr-reporting-summary-flat.pdf

Life sciences study design

All studies must disclose on these points even when the disclosure is negative.

Sample size	no sample size calculation was performed; approximate sample sizes of Comirnaty and Vaxzevria groups were determined a priori based on samples sizes from immunogenicity experiments of COVID-19 vaccines that had been analyzed with the same kind of assay (SARS-CoV-2 microneutralization assay; e.g. Walsh et al., N Engl J Med 2020;383:2439-50; Folegatti et al., Lancet 2020; 396: 467–78). The sample size of the NVX-CoV2373 group was then chosen similar to the Comirnaty group.
Data exclusions	No data were excluded from measurements.
Replication	Replicate measurements of the samples used in the study were not performed, as the underlying assay had previously been validated according to ICHQ2(R1) and only measurements with met predefined sample validity criteria, from assays with met predefined assay validity criteria, were subjected to further analyses.
Randomization	Not relevant, as the main goal of the study was to identify potential differences between distinct vaccines (for all of which the efficacy had already been proven in randomized clinical studies)
Blinding	Lab personnel were blinded as to the age and sex of the participants.

Reporting for specific materials, systems and methods

We require information from authors about some types of materials, experimental systems and methods used in many studies. Here, indicate whether each material, system or method listed is relevant to your study. If you are not sure if a list item applies to your research, read the appropriate section before selecting a response.

Materials & experimental systems

n/a	Involved in the study	
☒	☐	Antibodies
☐	☒	Eukaryotic cell lines
☒	☐	Palaeontology and archaeology
☒	☐	Animals and other organisms
☐	☒	Human research participants
☐	☒	Clinical data
☒	☐	Dual use research of concern

Methods

n/a	Involved in the study	
☒	☐	ChIP-seq
☒	☐	Flow cytometry
☒	☐	MRI-based neuroimaging

Eukaryotic cell lines

Policy information about cell lines

Cell line source(s)	European Collection of Authenticated Cell Cultures (ECACC); Cat.no. 84113001
Authentication	The cell line was obtained from an authenticated source (see above).
Mycoplasma contamination	Cell line was tested negative for mycoplasma contamination.
Commonly misidentified lines (See ICLAC register)	n.a.

Human research participants

Policy information about studies involving human research participants

Population characteristics	see Table 1 of the manuscript.
Recruitment	Participants were recruited from staff personell on a voluntarily basis (Comirnaty, Vaxzevria), or (for NVX-CoV2373) enrolled

Recruitment	in a phase 1/2 clinical study as described in study record detail of NCT04712110. All participants signed informed consent. All samples were de-identified starting from the original vials.
Ethics oversight	ethics committee of the Johannes Kepler University Linz (EC-No. 1322/2020)

Note that full information on the approval of the study protocol must also be provided in the manuscript.

Clinical data

Policy information about clinical studies

All manuscripts should comply with the ICMJE guidelines for publication of clinical research and a completed CONSORT checklist must be included with all submissions.

Clinical trial registration	NCT04712110
Study protocol	https://clinicaltrials.gov/ct2/show/NCT04712110
Data collection	Primary material (serum) was collected at Takeda occupational healthcare centers (Orth/Danube; Vienna), Med Campus III (University Clinic, Linz), Sumida Hospital (Sumida-ku, Tokyo, Japan), or Nishi Kumamoto Hospital (Kumamoto, Japan). Data was collected at Takeda Global Pathogen Safety (Vienna).
Outcomes	Primary outcome was pre-defined as SARS-CoV-2-neutralizing antibodies relative to WHO international standard 20/136.
